# Influence of Lifestyle Habits in the Development of Obesity during Adolescence

**DOI:** 10.3390/ijerph19074124

**Published:** 2022-03-31

**Authors:** Pedro Juan Carpena Lucas, Francisco Sánchez-Cubo, Manuel Vargas Vargas, José Mondéjar Jiménez

**Affiliations:** 1 Pediatric Service, Hospital Virgen del Castillo, 30510 Murcia, Spain; 2 Department of Political Economy and Public Finance, Economic and Business Statistics and Economic Policy, Faculty of Social Sciences, University of Castilla-La Mancha, 16007 Cuenca, Spain; francisco.scubo@uclm.es; 3 Faculty of Economic and Business Sciences, University of Castilla-La Mancha, 02006 Albacete, Spain; manuel.vargas@uclm.es; 4 Department of Statistics, Faculty of Social Sciences in Cuenca, University of Castilla-La Mancha, 16007 Cuenca, Spain; jose.mondejar@uclm.es

**Keywords:** importance-performance map analysis, lifestyle, prevention, structural equation modeling, teenagers, obesity, overweight

## Abstract

Background: The alarming increase in childhood obesity is a global public health problem since it has significant health consequences. This cross-sectional study aimed to identify the potentially modifiable risk factors for developing excess weight and determine the importance of developing certain habits to prevent childhood overweight and obesity. Methods: The sample included 416 students between the ages of 12 and 14 (12.8 ± 0.62) first-year high school students from Murcia in Spain. Data were collected on their lifestyle habits through the ENHASA questionnaire, and the somatometry of the participants was measured. Such data were studied through structural equation modeling and importance-performance map analyses. Results: The modifiable risk factors that presented the greatest potency directly regarding when developing excess weight in adolescents were ‘use of electronic devices’ and ‘not performing physical activity’ (*p* < 0.001). ‘Social and school environment’ and ‘diet’ showed relationships but no significant differences with overweight or obesity. Globally, the lifestyle habit of the greatest importance for not being overweight was ‘engagement in extracurricular physical activities’. On the other hand, the habits related to using new technologies in a sedentary way showed the best capacity for improvement. Therefore, it would be very efficient to focus on them to control excess weight. Conclusion: Responsible and limited use of screens and engaging in extracurricular physical activities may be the most remarkable and cost-effective strategies for obesity prevention programs.

## 1. Introduction

Obesity, a chronic non-communicable disease, is currently one of the main health problems in childhood as it is the most prevalent nutritional disorder among children these days [1,2]. It is not only a problem in westernized countries, but in recent years, it has become a global epidemic that must be addressed by public health authorities [3,4]. According to the World Health Organization (WHO) [3], in 2016, there were more than 340 million overweight or obese children, 10 million more than in 1990. These figures turn obesity into a global epidemic or pandemic [4,5]. As for Spain, according to the latest ALADINO study [6], more than a third of its child population is overweight, making it the European country with the highest number of overweight children [7], of which about half would eventually be obese adults [8].

Overweight and obesity in childhood have been associated with multiple health consequences. In the short term, the presence of obesity in childhood may imply the onset of cardiovascular diseases and type 2 diabetes at increasingly earlier ages [9,10,11,12,13,14,15,16], as well as orthopedic, gastrointestinal, metabolic, respiratory, neurological, or dermatological diseases [14,17,18,19,20,21,22,23,24,25], and last but not least, obesity can also cause sleep disorders in younger people, such as obstructive sleep apnea or insomnia [26,27], and psychological and social complications, such as depression, anxiety, or social isolation [28,29,30,31,32,33].

However, obesity in childhood also predisposes to mid and long-term health complications, such as coronary or cardiovascular disease or some types of cancer [8,19,31,34,35,36,37,38,39,40], and also the presence of inconveniences related to psychological health with a decrease in life expectancy [31,33,41,42,43]. Thus, for the first time in the history of humanity, there is a generation with lower quality of life than the previous one [44]. The aforementioned comorbidities also lead to increased health spending [45], which can be minimized by investing in obesity prevention programs from childhood.

Excess weight is a disease of multifactorial etiology in which genetic, psychosocial, family, and environmental factors intervene [17,46,47].

The control of the potentially modifiable risk factors for excess weight is currently the cornerstone of prevention and treatment [48,49]. Genetics, responsible for less than 5% of childhood obesity, must often be combined with contributing environmental and behavioral factors to affect weight [50]. So far, most of the foci have focused on poor eating habits and a sedentary lifestyle as the main causes of childhood obesity [5,51,52,53,54,55]. However, currently, the factors involved in obesity are many more, probably due to the social and cultural changes that have transpired in recent years that have promoted dramatic changes in people’s lifestyle habits. For one, the consumption of ultra-processed products has increased, and that of fruits, vegetables, and fish, which characterize the Mediterranean diet, has decreased [56,57,58]. That has produced an imbalance between energy intake and expenditure, contributing to a positive energy balance [19]. Moreover, using information and communication technology (ICT) has increased, and engagement in physical exercise has considerably decreased [59,60]. The direct dose-response relationship between heavy use of electronic media and increased BMI in children has already been well studied [42,61,62,63,64]. Regarding regularly practicing physical exercise, the positive relationship between children’s growth and the decrease in morbidity and mortality in adulthood is well known [17,65,66]. A sedentary lifestyle is associated with an increase in BMI in childhood, together with increased blood lipid levels, blood pressure, insulin resistance, and worsened bone mineralization. Conversely, not to forget the psychological benefits of regular exercise, such as self-confidence and self-esteem [56,58,67,68]. According to national studies, about 25% of the child–adolescent population is sedentary [6,57]. Due to this, the environment, particularly that to which young people are exposed, is increasingly obesogenic and will negatively influence the health of young people [69,70]. Regarding the environment, disadvantaged environments present higher rates of obesity in children, lower self-esteem, and worse scores on quality of life scales [30,71,72]. Moreover, children who are overweight or obese tend to have lower self-esteem and more depressive symptoms [30,72,73]. Moreover, some researchers point out that adolescents with better eating habits may have better physical aptitudes [74] or that the greater usage of ICT may be related to less physical exercise [60,75]. Hence, it is crucial to determine which modifiable lifestyle factors determine the development of excess weight in young people to a greater extent than the others do and if they do so jointly.

The objective of this study was to determine if certain lifestyle habits have a causal relationship with the development of excess weight in adolescence. As a secondary objective, an importance-performance analysis on lifestyle habits aimed to determine the lifestyle habits with the largest capacity for improvement.

For this, with previously developed bibliographic support, the following hypotheses were formulated:

**Hypothesis** **1** **(H1).** *Worse dietary factors positively influence the development of excess weight.*

**Hypothesis** **2** **(H2).** *An unfavorable social and school environment positively influences the development of excess weight*.

**Hypothesis** **3.1** **(H3.1).** *The use of information and communication technology associated with a sedentary lifestyle (“ICT-1”) positively influences the development of excess weight*.

**Hypothesis** **3.2** **(H3.2).** *The use of information and communication technology not associated with physical inactivity (“ICT-2”) is positively related to the development of excess weight*.

**Hypothesis** **4** **(H4).** *Less physical activity has a positive influence on the development of excess weight*.

## 2. Materials and Methods

This study was a cross-sectional study that examined 12- to 14-year-old students (i.e., in their first year of secondary education) from Area V of the Region of Murcia in Spain during the 2017–2018 and 2018–2019 school years. Using data from the National Institute of Statistics, a minimum sample size of 350 students was determined, with a maximum error of 4.92% and a 95% confidence interval. First, all secondary schools in the area were invited to participate in the study. Five out of them (four public and one private) agreed to do it. The rest of the schools declined to participate in the study, referring to difficulties with scheduling or lack of time. Since several schools refused to participate, a convenience sample was required instead of a randomized one, respecting the proportionality of the centers and obtaining a final sample larger than the minimum needed. The corresponding ethics committee authorized the study.

Data were collected in two phases. Firstly, information about children’s life habits was collected through the validated ENHASA questionnaire (Encuesta de Hábitos Saludables en Adolescentes) [76], which is a questionnaire that analyses the main modifiable behaviors that can lead to the development of childhood obesity. The questionnaire consisted of 26 items distributed among four dimensions using a 0-to-10-point Likert scale. The items of the questionnaire were distributed in the following four dimensions:

Dietary factors (D). Eight items that assess the following eating patterns: (D-1) They bring lunch from home, (D-2) they have at least four meals a day, (D-3) they eat fruit every day, (D-4) they eat vegetables or salads every day, (D-5) they eat fish several times a week, (D-6) they eat legumes several times a week, (D-7) they eat processed or fast food (pizza, hamburgers…) several times a week, and (D-8) they eat together with the family.

Environment factors (EN). The social and school environment are assessed with the following seven items: (EN-1) They have been bullied or threatened by other children, (EN-2) they are scared of other children, (EN-3) they are ashamed of themselves or would like to change a part of their body, (EN-4) they have felt everything goes wrong, (EN-5) they have felt lonely, (EN-6) they have complained that their parents do not have enough time for them, and (EN-7) they have complained that they are unfairly treated at home.

Information and communication technologies factors (ICTs). It contains seven items divided into two subsections. One that assesses compulsorily sedentary habits: (ICTs-1) They watch TV, (ICTs-2) they use a computer, mobile phone, or tablet to play video games, and (ICTs-3) they have a total screen time of 2 or more hours a day. There is another block that assesses the use of technology that is not necessarily sedentary through the following items: (ICTs-4) They use the computer, mobile phone, or tablet to chat with friends, (ICTs-5) they use the computer, mobile phone, or tablet to go online, (ICTs-6) your child gets angry if someone bothers them while using their mobile phone, and (ICTs-7) you have told someone that your child spends too much time using the phone or watching TV.

Physical activity (PA). It was analyzed with the following four items: (PA-1) They do some kind of exercise every day for at least 60 min, (PA-2) they do some extracurricular sports, such as tennis, dance, football, etcetera, (PA-3) they do physical activities with their family, e.g., walking, riding a bike, hiking, etcetera, and (PA-4) they have enough time for leisure activities: Playing, reading, etcetera.

A copy of this questionnaire was given to the parents of the students to be filled out at home. Besides, they received an information sheet and an informed consent form. Along the next month, the collaborating teachers collected the questionnaires and the duly completed informed consent forms for participating in the study.

A month later, in the second phase of the study, the health team traveled to the participating schools to determine the students’ body measurements. For this, the students were barefoot and in light clothing. The students’ weights, heights, and abdominal circumference were measured using the SECA^®^ 778 column scale with a built-in height rod (min. 2 kg and max. 200 kg) and the SECA^®^ 201 tape measure with precision of up to 1 mm, respectively. All the anthropometric measurements were conducted by the same person twice, recording the mean values.

The study participants were classified according to (1) their body mass index (BMI), based on the z-scores for sex and age, following WHO’s international references [77], and (2) their waist-to-height ratio (WHtR) and waist-to-hip ratio (WHR), using the cut-off points established according to sex and age [78,79]. Based on these data, the study participants were classified into the normal weight group and the excess weight group, being the latter one which includes the overweight or obese participants.

Before the statistical analysis, through the effect size and the power tables [80] and using Green’s approximation [81] for five predictors and the medium effect size *n* ≥ 91, the sample was verified to be sufficient for the execution of the models. Subsequently, two analyses were performed to study the relationships between certain lifestyle habits and weight status. First, a model was designed using the partial least squares (PLS) method to measure the intensity of the relationships between the indicators and the constructs. Then bootstrapping was executed with 10,000 samples, which allowed the contrast of the formulated hypotheses. Finally, importance-performance map analysis (IPMA) was performed to determine the importance and relative performance of the latent variables considered. Items with >0.7 correlation values, with *t*-value (degree of significance for each dimension) >2.575 (99% confidence) and with final R^2^ > 0.1 were accepted. The modeling was done using the SmartPLS 3.0 statistical software (IBM, Armonk, NY, USA).

## 3. Results

From the population of 561 eligible students, 74.2% participated in the study, 48% were men, with a mean age of 12.8 ± 0.62 years. Among those who participated in the study, five were excluded because they presented pathologies that could interfere with their nutritional status: Turner syndrome, uncontrolled hypothyroidism, trichorhinophalangeal syndrome, maturity-onset diabetes of the young, or reduced mobility. Thus, the final study sample consisted of 416 participants. Regarding their BMI, excess weight was observed in 40.6% of them. By their WHtR, 35.1% of them and by their WHR, 39.2%. There were no significant differences between the sexes.

The multivariate analysis presented a final R^2^ of 0.109, confirming the explanatory power of the model. The individual reliability study of the indicators showed values above those recommended (Table 1).

**Figure 1:**
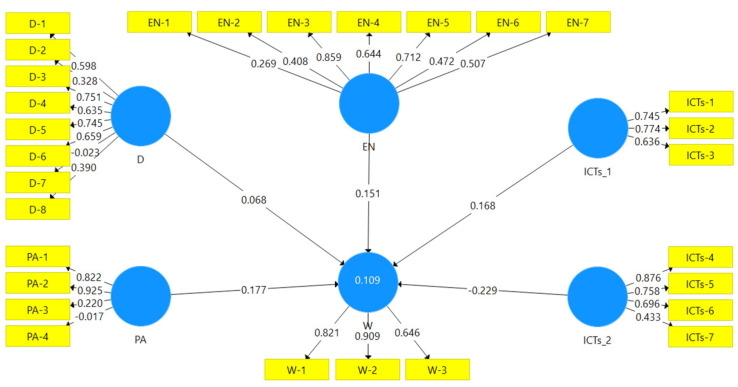
General results of the structural equations model. D: Dietary factors; EN: Environment; ICTs_1: Sedentary information and communication technologies; ICTs_2: Information and communication technology not associated with physical inactivity; PA: Physical activity; W-1 (BMI): Body Mass Index; W-2 (WHtR): Waist to height ratio; W-3 (WHR): Waist to hip ratio.

The main results are presented in Figure 1. They show a direct and positive relationship between each construct and excess weight, except for ICT_2, which has a negative relationship, and they show that the physical activity and ICT factors have the highest magnitudes.

Regarding the contrast of the hypotheses through bootstrapping (Table 2), only one hypothesis has a non-significant relationship: H1. However, the rest of the hypotheses are statistically significant, so they are supported.

Table 3 shows the loadings of each item of the ENHASA questionnaire within the construct they form.

For H1, the habits with the highest potency were those related to the intake of fruits, vegetables, and fish. For H2, the obtained value did not show a significant association at 99%, although it did at 90% (*p* = 0.082), again supporting the hypothesis. The most important variable in this construct was the one referring to whether the child is ashamed of him/herself. As for H3.1 and H3.2 (ICT_1), it showed a magnitude of 0.168 and a 2.666 *t*-value (statistically significant), supporting the hypothesis. The most significant item was related to the use of video games. H3.2 (ICT_2) showed a significant negative relationship with the development of excess weight, thus supporting the hypothesis. The variable with the greatest weight was the use of technology to contact friends. Finally, for H4 concerning physical activity, a direct and significant relationship was found with the development of excess weight, with a 0.177 magnitude and a 2.678 *t*-value, supporting the hypothesis. Consequently, the habit with the greatest potency is engagement in extracurricular physical activities.

**Figure 2:**
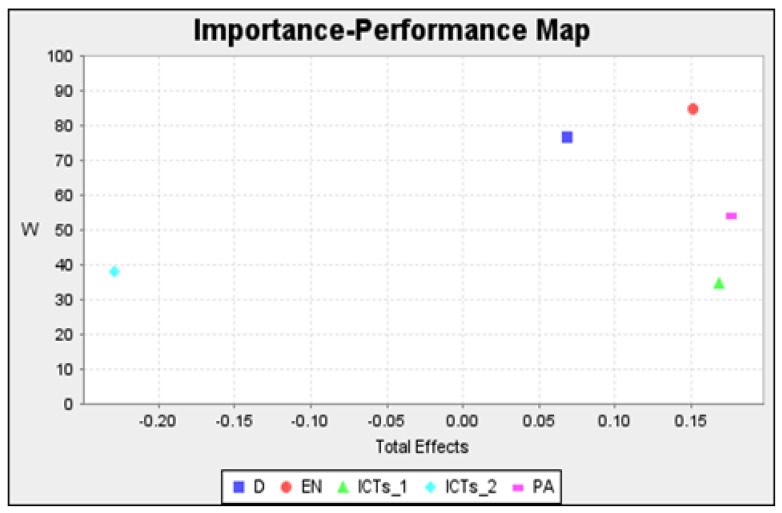
Importance-performance map of constructs to weight. D: Dietary factors; EN: Environment; ICTs_1: Sedentary information and communication technologies; ICTs_2: Information and communication technology not associated with physical inactivity; PA: Physical activity; W: Weight.

Regarding IPMA, Figure 2 presents the constructs in a two-dimensional grid that considers their importance and performance.

**Figure 3:**
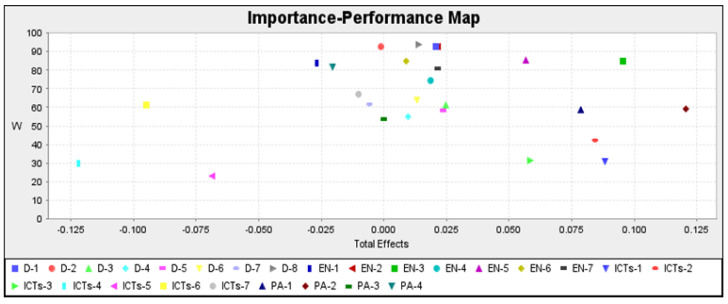
Representation of each life habit assessed in the ENHASA questionnaire (see Table 3). Importance-performance map of latent variables to weight.

In general, all the dimensions had acceptable values. The most important constructs were PA and ICT-1, the latter presenting a relatively low performance (close to 55% and 35%, respectively). In the individual analyses of the lifestyle habits (Figure 3), it was found that the most important lifestyle habit overall was engagement in extracurricular physical activities, and the lifestyle habit with the greatest capacity for improvement was the use of new technologies associated with a sedentary lifestyle, with values close to 40%. The critical areas of care and intervention were thus identified.

## 4. Discussion

According to the data obtained based on the WHO parameters, approximately two in five young people in the current study has excess weight, mirroring the figures obtained from national and international studies [6,82,83,84]. Given the well-known relationship between central obesity and alterations in the lipid profile and metabolic syndrome [34,35], we decided to include the WHtR and WHR not to underestimate the impact of the risk factors, which present a greater correlation with infantile adiposity [19,36].

The relationship between lifestyle habits and the development of excess weight in adolescence is a widely studied topic [3,49,51,55]. However, the current study used the novel partial least squares structural equation modeling (PLS-SEM) method, which is widely used in other fields and whose results allow the hypotheses to be contrasted and actions in certain areas susceptible to improvement to be proposed [20,36,37].

Regarding the first hypothesis (H1), no significant relationship was found between dietary factors and obesity, like other studies [2,49,56,85]. The results were found in the present study probably because the study sample belonged to a rural area, in which young people maintain good adherence to the Mediterranean diet, which is rich in fruits, vegetables, and fish [86]. This phenomenon is also observed in other Mediterranean countries [86,87,88]. Adherence to the Mediterranean diet has been proven to decrease mortality, particularly from cardiovascular diseases, and the incidence of other chronic diseases [87,88]. Thus, living in a rural area may imply protective eating patterns for health.

Concerning the social and school environment (H2), it should be noted that this dimension did not show a 99% significance, but it was a 90% that confirms its importance. It must be considered that adolescence is a stage of vulnerability in which feelings of self-esteem, self-criticism, and personal satisfaction arise. Higher rates of obesity and poor self-esteem and quality of life are shown in disadvantaged environments [30,71,72], so it is crucial to create a comfortable environment both at the social and school levels [30,43,71,72,89].

The use of electronic devices (H3.1 and H3.2) was the most relevant dimension in determining the development of overweight and obesity in adolescents. Given the demonstrated importance of this construct, it was divided into two subgroups to conduct a more exhaustive analysis: ICT-1, made up of items associated with sitting, and ICT-2, made up of others not associated necessarily with physical inactivity. Leisure, education, or communication through a screen has had a sharp rise among adolescents, and a direct relationship was found between the intensive use of such media and the increase in BMI in childhood [49,61,62,63]. The negative relationship between ICT-2 and the development of excess weight in childhood may be explained by parents’ incorrect perception of the usage of new technologies, which makes it difficult for them to identify when the use of such technologies is already abusive [75,90]. Besides, IPMA showed that the items that constituted sedentary habits presented a performance lower than 50%, thus demonstrating the lifestyle habits that would allow greater obtained improvement with intervention.

The last hypothesis (H4), related to exercise, was also demonstrated to have a significant and direct relationship with excess weight. The PA was previously identified as the key to maintaining a healthy weight [56,58,91]. The IPMA showed that the most important lifestyle habit for preventing the development of excess weight was engagement in extracurricular physical activities such as sports, which coincides with the result of the ALADINO study [6]. Therefore, promoting and facilitating engagement in these activities should be a priority, and attempts should be made to overcome the socioeconomic or geographic factors that make access to them difficult.

The strengths of the current study are (1) the use of a validated questionnaire for the pediatric population and (2) the objective method that was used to obtain somatometric data, using standardized protocols, which reduce biases. Additionally, the current study applied a novel statistical technique in this field of study. As for the weaknesses of the current study, it outstands the recruitment of participants from a single health area, making it impossible to extrapolate the results to other population sets. Plus, randomization by convenience is identified as a disadvantage. There could also be data bias as these were obtained from a self-declared questionnaire. Nevertheless, it is a simple questionnaire, easy to apply and validated in the same age range. It could present certain biases derived from problems in the wording of the questions, problems with the design and layout of the questionnaire or derived from problems with the use of the questionnaire. Finally, most of the surveys were completed by a single progenitor (especially mothers). Some works affirm that the answers in the quality-of-life questionnaires can differ depending on the parent who responds.

## 5. Conclusions

In this piece of work, the main risk factors related to the development of obesity during adolescence were studied. The use of new technologies and engagement in physical activities were identified as the most determining factors. On the other hand, it was shown that sedentary habits, such as watching television, playing video games, or spending more than 2 h in front of a screen, have the greatest capacity for improvement to avoid child obesity. Public institutions should promote limited and responsible screen time, in addition to improving the accessibility of engaging in extracurricular physical activities such as sports. The aim of these is to achieve a shift in the present situation in which the current proportions of time spent by adolescents on-screen and engaging in physical activities are reversed.

The model proposed and validated in this study can be used by institutions to design and initiate more effective child obesity prevention and intervention programs.

## Figures and Tables

**Table 1:** Reliability measures and constructs validity.

	Cronbach’s Alpha	rho_A	Composite Reliability	Average Variance Extracted (AVE)	R-Square
dietary factors	0.684	0.707	0.754	0.322	
environment	0.759	0.677	0.764	0.340	
ICTs_1	0.544	0.556	0.763	0.519	
ICTs_2	0.696	0.750	0.793	0.503	
physical activity	0.534	0.728	0.611	0.395	
weight	0.712	0.767	0.839	0.640	0.109

ICTs_1: Sedentary information and communication technologies. ICTs_2: Information and communication technology not associated with physical inactivity.

**Table 2:** Hypothesis tests between constructs and excess weight.

	Original Sample (O)	Sample Mean (M)	Standard Deviation (STDEV)	T Statistics (|O/STDEV|)	*p* Values
D -> W	0.068	0.106	0.059	1.164	0.245
EN -> W	0.151	0.160	0.087	1.737 ^b^	0.082
ICTs_1 -> W	0.168	0.153	0.063	2.666 ^a^	0.008
ICTs_2 -> W	−0.229	−0.213	0.073	3.133 ^a^	0.002
PA -> W	0.177	0.175	0.066	2.678 ^a^	0.007

^a^ Values with significance level at 0.01. ^b^ Values with significance level at 0.10. D: Dietary factors; EN: Environment; ICTs_1: Sedentary information and communication technologies; ICTs_2: Information and communication technology not associated with physical inactivity; PA: Physical activity; W: Weight.

**Table 3:** ENHASA questionnaire and weighting factor of each item post multivariate analysis.

		Indicator	Standardized Factor Weighting
dietary factors		D-1 Brings lunch from home.	0.598
		D-2 Has at least four meals a day.	0.328
		D-3 Eats fruit every day.	0.751
		D-4 Eats vegetables or salads every day.	0.635
		D-5 Eats fish several times a week.	0.745
		D-6 Eats legumes several times a week.	0.659
		D-7 Eats processed or fast food (pizza, hamburgers…) several times a week.	−0.023
		D-8 Eats together with the family.	0.390
environment		EN-1 Has been bullied or threatened by other children.	0.269
		EN-2 Is scared of other children.	0.408
		EN-3 Is ashamed of themselves or would like to change a part of their body.	0.859
		EN-4 Has felt everything goes wrong.	0.644
		EN-5 Has felt lonely.	0.712
		EN-6 Has complained that their parents do not have enough time for them.	0.472
		EN-7 Has complained that they are treated unfairly at home.	0.507
information and communication technologies (icts)	ICTs_1	ICTs-1 Watches TV.	0.745
		ICTs-2 Uses a computer, mobile phone, or tablet to play video games.	0.774
		ICTs-3 Has a total screen time of 2 or more hours a day.	0.636
	ICTs_2	ICTs-4 Uses the computer, mobile phone, or tablet to chat with friends.	0.876
		ICTs-5 Uses the computer, mobile phone, or tablet to go online.	0.758
		ICTs-6 Your child gets angry if someone bothers them while using their mobile phone.	0.696
		ICTs-7 You have told someone that your child spends too much time using the phone or watching TV.	0.433
physical activity		PA-1 Does some type of exercise every day for at least 60 min.	0.822
		PA-2 Does some extracurricular sports activities such as tennis, dance, football, etc.	0.925
		PA-3 Does physical activities with their family, e.g., walking, riding a bike, hiking, etc.	0.220
		PA-4 Has had enough time for leisure activities: playing, reading, etc.	−0.017

*n* = 416. ICTs-1: Sedentary information and communication technologies. ICTs-2: Information and communication technology not associated with physical inactivity.
